# A Web-Based Physical Activity Promotion Intervention for Inactive Parent-Child Dyads: Protocol for a Randomized Controlled Trial

**DOI:** 10.2196/55960

**Published:** 2024-03-21

**Authors:** Daniel Phipps, Weldon Thomas Green, Reetta Aho, Eeva Kettunen, Stuart Biddle, Kyra Hamilton, Arto Laukkanen, Kaisa Aunola, Derwin King Chan, Nelli Hankonen, Mary Hassandra, Tommi Kärkkäinen, Virpi-Liisa Kykyri, Juho Polet, Ryan Rhodes, Montse C Ruiz, Arja Sääkslahti, Jekaterina Schneider, Hanna-Mari Toivonen, Taru Lintunen, Martin Hagger, Keegan Knittle

**Affiliations:** 1 Faculty of Sport and Health Sciences University of Jyväskylä Jyväskylä Finland; 2 School of Applied Psychology Griffith University Brisbane Australia; 3 Centre for Health Research University of Southern Queensland Brisbane Australia; 4 Health Sciences Research Institute University of California - Merced Merced, CA United States; 5 Department of Psychology University of Jyväskylä Jyväskylä Finland; 6 Department of Early Childhood Education Education University of Hong Kong Hong Kong China (Hong Kong); 7 Faculty of Social Sciences Tampere University Tampere Finland; 8 Department of Physical Education and Sport Science University of Thessaly Thessaly Greece; 9 Centre of Excellence in Learning Dynamics and Intervention Research (InterLearn) University of Jyväskylä and University of Turku Jyväskylä Finland; 10 School of Exercise Science, Physical and Health Education University of Victoria Victoria, BC Canada; 11 Centre for Appearance Research School of Social Sciences University of the West of England Bristol United Kingdom; 12 Department of Psychological Science University of California - Merced Merced, CA United States

**Keywords:** dyadic behavior change, family behavior change, intervention, physical activity, theory of planned behavior

## Abstract

**Background:**

Low levels of physical activity are associated with numerous adverse health outcomes, yet sedentary lifestyles are common among both children and adults. Physical activity levels tend to decline steeply among children aged between 8 and 12 years, even though children’s behavioral patterns are largely governed by familial structures. Similarly, parents’ activity levels have been generally reported as lower than those of nonparents of comparable age. For this reason, family-based physical activity promotion interventions are a potentially valuable and relatively underresearched method for mitigating physical activity declines as children develop into adolescents and for increasing physical activity in parents.

**Objective:**

This study aims to assess the efficacy, feasibility, and acceptability of a novel theory-based web-based physical activity promotion intervention among parent-child dyads in Finland who do not meet physical activity recommendations at baseline.

**Methods:**

Participants (target N=254) will be recruited from the general population using a panel company and advertisements on social media and randomly assigned to either an immediate intervention group or a waitlist control group. The intervention consists of 4 web-based group workshops over the course of 10 weeks, web-based tasks and resources, and a social support chat group. Data on physical activity behavior and constructs from the integrated behavior change model will be collected through self-report surveys assessing physical activity, autonomy support, autonomous motivation, attitude, subjective norm, perceived behavioral control, intention, self-monitoring, habit, and accelerometer measurements at baseline, post intervention, and 3 months post intervention. Exit interviews with participants will assess the feasibility and acceptability of the intervention procedures.

**Results:**

This study will reveal whether the intervention changes leisure-time physical activity among intervention participants relative to the control group and will examine the intervention’s effects on important theoretical predictors of physical activity. It will also yield data that can be used to refine intervention materials and inform further implementation. Trial recruitment commenced in September 2023, and data collection should be completed by December 2024.

**Conclusions:**

The planned intervention has potential implications for both theory and practice. Practically, the use of an entirely web-based intervention may have scalable future uses for improving physical activity in 2 key populations, while also potentially informing on the value of dyadic, family-based strategies for encouraging an active lifestyle as an alternative to strategies that target either parents or children independently. Further, by assessing change in psychological constructs alongside potential change in behavior, the intervention also allows for important tests of theory regarding which constructs are most linked to favorable behavior change outcomes.

**Trial Registration:**

ClinicalTrials.gov NCT06070038; https://clinicaltrials.gov/study/NCT06070038

**International Registered Report Identifier (IRRID):**

DERR1-10.2196/55960

## Introduction

### Overview

Low levels of physical activity in adult and youth populations are associated with an increased risk of physical and mental health conditions and a reduced quality of life. Conversely, regular physical activity participation is associated with reduced chronic disease risk and better psychological health and well-being [1]. Accordingly, the World Health Organization has published evidence-based guideline levels of physical activity required to realize these health benefits. The guidelines recommend that adults aged between 18 and 64 years participate in at least 75 minutes of vigorous physical activity, 150 minutes of moderate physical activity, or an equivalent combination of both per week, while children aged between 7 and 17 years are recommended to participate in at least 60 minutes of physical activity per day. Studies indicate that most people do not achieve these guideline levels of physical activity [2]. Further, studies have observed a sharp drop in physical activity participation in child and adolescent populations, followed by generally low participation levels into and throughout adulthood [3].

Given the steep decline in activity levels, as children transition into adolescence, the development and implementation of behavioral interventions to encourage an active lifestyle is a key target area for health promotion research. One strategy proposed to enhance the efficacy of behavior change strategies for children’s activity levels is to target the family unit rather than children themselves. Specifically, parents of preteen children retain a strong influence on their child’s behavior, both through their own activity levels [4,5] and through the opportunity to provide support for and foster motivation toward leisure-time physical activity behaviors [6,7]. Further, evidence indicates that children likely influence physical activity behaviors and beliefs in their parents, as parents tend to be less active than nonparents [8], often citing their children’s lack of motivation or support as a barrier to being physically active [9]. This evidence of within-family effects indicates the potential utility of dyadic interventions for both parents and children [10], using theory-driven, group-based behavior change strategies to bolster social support, foster motivation, and reduce the perceived barriers to behavior in both the parent and child. Yet, despite evidence for the potential utility of these strategies, few physical activity interventions have been applied for parent-child dyads, and those that have tend not to have a strong basis in behavioral theory and are seldom evaluated systematically [11], inhibiting meaningful conclusions on their efficacy. In response to the relative scarcity of theory-driven, family-based physical activity intervention programs, we aim to develop and test an intervention to promote physical activity in low-active parents and their children based on the integrated behavior change model, an approach that outlines the multiple determinants and potential targets for intervention derived from multiple theoretical perspectives [12].

### The Integrated Behavior Change Model

The integrated behavior change model draws from several well-established behavioral theories: self-determination theory [13], the theory of planned behavior [14], the health action process approach [15], and the reflective impulsive model [16]. Central to the model is that individuals’ quality of motivation, which reflects whether their behavior is consistent with the self-endorsed reasons, is highly influential in individuals’ intentions to perform physical activity and physical activity participation. This premise is derived from self-determination theory, which makes the distinction between autonomous and controlled forms of motivation. Autonomous motivation reflects an individual’s performing physical activity consistent with their own interests, choices, needs, and sense of personal involvement [13,17]. By contrast, controlled motivation reflects performing activities for externally referenced reasons, such as for rewards or out of obligation to others. Of critical importance when it comes to performing physical activity, individuals who perform physical activities for autonomous motives are more likely to form intentions to perform physical activity in the future and are more likely to develop routines and habits, which can translate to long-term physical activity persistence [18]. This is because those citing autonomous reasons for performing physical activity are likely to persist because their motivation emanates from themselves, while those whose motives are controlled will only persist as long as the external contingencies (eg, rewards and demands from others) persist. A key tenant of self-determination theory [17,19], is that autonomous motivation for physical activity can be fostered through the support of salient others, such as parents or teachers. For example, parents who display behaviors that indicate support for children’s autonomy and competence toward physical activity and demonstrate a sense of unconditional relatedness with their children for physical activities, are more likely to foster autonomous motives in their children toward performing physical activity in the future [6,7,20]. As such, enabling parents to display autonomy-supportive behaviors with respect to presenting, discussing, and performing physical activity with their children is likely an important strategy to promote physical activity participation in children and may be particularly valuable in family-based interventions aimed at promoting physical activity. It is also likely to be useful in parent-child dyads, where both parent and child can be encouraged to display behaviors that support each other’s autonomous motivation. This is supported in empirical data, where autonomy-supportive parenting has been associated with autonomous motivation in children as well as positive behavioral outcomes [21], including enhanced physical activity [6].

The integrated behavior change model also specifies the processes by which autonomous motivation leads to intention toward, and actual participation in, physical activity in the future. Specifically, individuals who are autonomously motivated toward a behavior are proposed to be more likely to form adaptive beliefs in favor of performing that behavior in the future [12,22-24] and, as a consequence, form an intention to perform physical activity in the future. Such beliefs are represented in the model by the belief-based attitude, subjective norm, and perceived behavioral control constructs from the theory of planned behavior [14], a prototypical theory that identifies the antecedents of intentional behaviors such as physical activity. The theory stipulates intention is the salient predictor of subsequent behavior, and intentions themselves are a function of attitudes (beliefs about the perceived likely affective or instrumental outcomes of engaging in a behavior), subjective norms (beliefs about where important others in one’s life would want them to engage in a behavior or not), and perceived behavioral control (beliefs about whether engaging in a behavior is under one’s own control or within their abilities). Research has demonstrated that these beliefs tend to be reliably related to physical activity intentions and participation, signaling their potential as modifiable constructs that could be targeted in intervention strategies aimed at promoting positive intentions toward, and actual participation in, physical activity. Accordingly, interventions based on the theory and targeting the belief-based constructs have shown efficacy in changing intentions and behavior [25]. For example, interventions presenting persuasive messages that highlight the advantages of behavior and downplay the disadvantages, targeting attitude change, or prompting practice that assists individuals in successfully mastering the target behavior and overcoming obstacles, targeting perceived behavior control change, have been shown to be effective in promoting intention and behavior change in physical activity contexts [26].

While there is evidence for the utility of interventions based on the recommendations of self-determination theory (eg, use of strategies like fostering autonomy support in influential others) or the theory of planned behavior (eg, providing persuasive communications targeting belief change), the integrated behavior change model also acknowledges that these strategies are often more efficacious in changing motivation or intention than changing behavior [27]. Recognizing the shortfall in the association between motivation and behavior, such as relatively modest intention-behavior relations observed in physical activity [28-32], other strategies that bolster intentions may be useful. For example, researchers adopting action control frameworks have suggested that leveraging [28,33] intervention strategies such as planning and self-monitoring may strengthen the intention-behavior relationship and increase the likelihood that individuals act on their good intentions when performing physical activity [15,34].

### Study Overview Objectives

Given the need for interventions to help children maintain physical activity levels as they transition into adolescence and to help parents become more physically active, our group used the integrated model as a starting point to develop a novel, remotely delivered dyadic physical activity promotion intervention. By applying the integrated behavior change model to a parent-child intervention, we aim to use and strengthen within-family dynamics to foster autonomous motivation and encourage physical activity in both parents and preteen children. In this protocol, we describe a planned randomized controlled trial for testing the effects of this intervention in a sample of insufficiently active Finnish-speaking parents and children.

## Methods

### Trial Design

The trial will adopt a randomized waitlist control design in which parent-child dyads are the unit of randomization. Families will be randomized on sign-up to either an intervention group that receives the intervention immediately after baseline data collection or to a waitlist control group that will receive the intervention after all outcome data have been collected.

### Participant Recruitment and Eligibility

Parent-child dyads will be recruited from the general Finnish population through direct contact with a panel company, through social media advertisements, and through posts on parenting discussion boards and forums. Individuals who responded to the advertisements were directed to a screening survey hosted on the Webropol platform. To be eligible for inclusion, dyads must consist of a parent or guardian aged >18 years and a child aged 8-12 years, and both dyad members need to be considered sedentary. Parents are considered sedentary if they were not active for at least 30 minutes a day on 5 or more days in the past week, and children are considered sedentary if they were not active for at least 60 minutes per day in the past week. Dyads will be excluded if either the parent or child reported having a medical condition or injury likely to prevent them from safely engaging in physical activity. People meeting the inclusion criteria can continue in the Webropol survey to read information about the trial and are then offered the opportunity to provide their contact details to opt-in to the trial.

### Power Analysis

The projected sample size was estimated from a statistical power analysis conducted using G*Power (version 3.1; Heinrich-Heine-Universität Düsseldorf). The analysis assumed a small to medium effect size (Cohen *f*=0.235) for the intervention on our primary outcome variable, leisure time physical activity, calculated from an average of effect sizes from meta-analyses of self-determination theory–based interventions (Hedges *g*=0.45 [35]) and interventions targeting self-efficacy (Cohen *d*=0.48 [36]), with α set at .05 and statistical power set at .80 reveal a projected total sample size of 178 participants. We also assumed a projected 30% attrition rate in participants through the study based on similar trials [37], resulting in a sample of 254 participants (ie, 127 dyads) to be recruited at baseline.

### Study Procedures

All recruitment materials will be delivered in a web-based format with a URL forwarding participants to a screening and informed consent survey hosted on the Webropol platform. First, parents will be presented with an eligibility questionnaire to assess whether they and their children making reference to themselves and their least active child aged between 8 and 12 years, meet the inclusion criteria. Eligible parents will then be presented with information about the study and an informed consent form. Parents who provide informed consent to participate in the study will be prompted to provide their name, contact details, and the name of their least active child, aged between 8 and 12 years, and will be enrolled in the study.

After enrolling in the trial, dyads will be assigned by computerized random-digit generation to 1 of 2 groups: an immediate intervention group that receives the intervention immediately after baseline data collection, or a waitlist control group that will receive the intervention after all outcome data have been collected.

Participants in both groups will be asked to provide outcome data immediately after randomization, 3 months later, and 6 months later. Outcome data will be collected through web-based surveys hosted on the Webropol platform and through physical activity measurement devices mailed to participants’ homes. For web-based surveys, parents will be emailed 2 separate URLs: 1 for themselves and 1 for their child. Parents will be instructed that they may help their child understand the survey items but should avoid influencing their child’s answers to the questionnaire.

**Figure 1:**
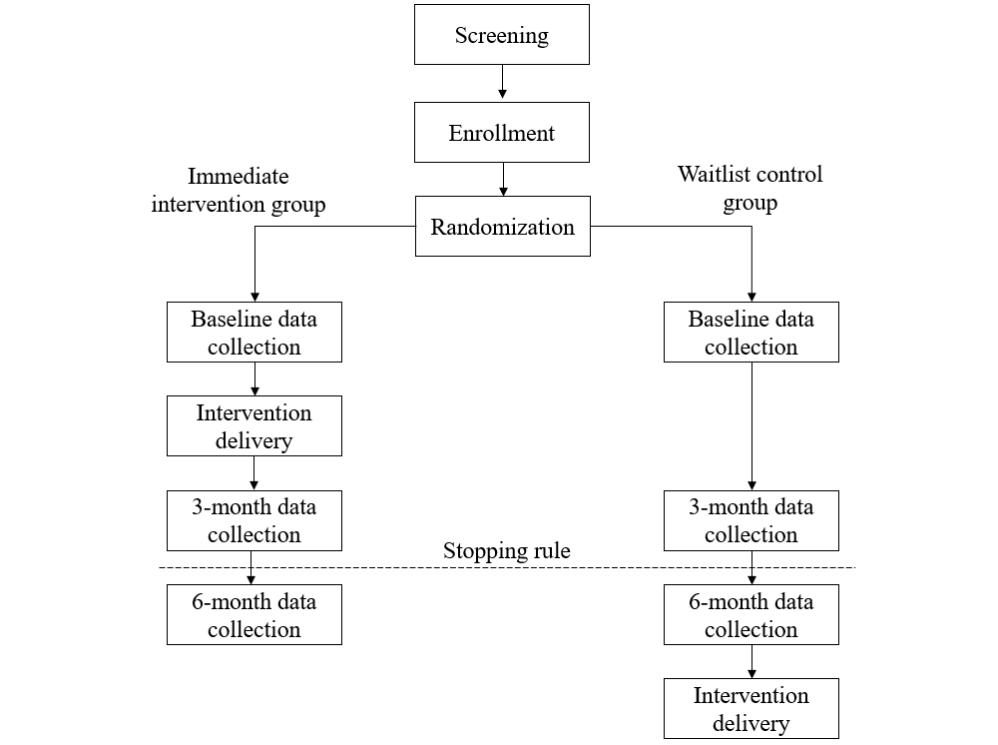
A flowchart of enrollment and data collection for the ProAct trial.

Participants in the immediate intervention group will receive the intervention between the baseline and 3-month data collection points, while participants in the waitlist control group will receive the intervention after all data collection has been completed. While data are planned to be collected at baseline, 3 months, and 6 months, the trial includes a stopping rule, such that 6-month follow-up data will not be collected if there is no effect of the intervention on the primary outcome (ie, leisure time physical activity) at the 3-month follow-up. The trial design flow diagram is presented in Figure 1.

### Intervention

#### Immediate Intervention Group

The intervention consists of 4 web-based sessions hosted through a videoconferencing platform (Zoom Inc) and facilitated by 1 or 2 members of the research team. The 4 sessions will be delivered in weeks 1, 3, 5, and 8 of the program. Summaries of each session, including the target constructs and behavior change techniques used in each session, are presented in Table 1. The first session will involve only parents and will last 90 minutes. It focuses on instructing parents in the use of autonomy-supporting parenting behaviors. The remaining 3 sessions will involve both parents and children and will last 45 minutes. Session 2 prompts a discussion of the benefits of an active lifestyle, including enjoyment and importance, and gets participants to set individual and joint physical activity-related goals. Session 3 teaches participants how to make action plans and coping plans (ie, problem-solving) when pursuing physical activity goals. Session 4 covers social norms related to physical activity, has participants specify their identity related to physical activity, and prompts social support strategies within each dyad.

**Table 1:** Content and targets for each intervention session in the ProAct trial. Behavior change techniques preceded by an “M” are drawn from the Motivation and Behavior Change Techniques [38]. Behavior change techniques preceded by a “T” are drawn from the Behavior Change Taxonomy version 1 [39].

Session	Target constructs	Behavior change techniques
Session 1: 90 minutes and parents only	Autonomy supportive parenting	M3. Use non-controlling, informational language M5. Provide a meaningful rationale M6. Provide choice M7. Encourage the person to experiment and self-initiate the behavior M10. Show unconditional regard M12. Use empathic listening M16. Clarify expectations T1.1 Goal setting yeah T1.4 Action planning T4.1 Instruction on how to perform the behavior T4.2 Information about antecedents T5.3 Information about social and environmental consequences T5.6 Information about emotional consequences T8.1 Behavioral practice/rehearsal T16.3 Vicarious consequences
Session 2: 45 minutes, parents, and children	Attitude	M4. Explore life aspirations and values M6. Provide choice M7. Encourage the person to experiment and self-initiate the behavior M16. Clarify expectations M17. Assist in setting optimal challenge M19 Help develop a clear and concrete plan of action M20 Promote self-monitoring T1.1 Goal Setting (behavior) T1.3. Goal Setting (outcome) T1.5 Review behavior goal(s) T1.6 Discrepancy between current behavior and goal T2.3 Prompt self-monitoring of behavior T4.1 Instruction on how to perform the behavior T5.6 Information about emotional consequences T15.3 Focus on past success
Session 3: 45 minutes, parents, and children	Perceived behavioral control and self-regulation	M15. Address obstacles for change M19. Help develop a clear and concrete plan of action M20. Promote self-monitoring M21. Explore ways of dealing with pressure T1.2 Problem-solving T1.5 Review behavioral goals T1.6. Discrepancy between current behavior and goal T2.2 Feedback on behavior T8.7 Graded Tasks T15.1 Verbal persuasion about capability T15.3 Focus on past success.
Session 4: 45 minutes, parents, and children	Subjective Norm and perceived autonomy support	M2 Prompt identification of sources of pressure for behavior change M8. Acknowledge and respect perspectives and feelings M9. Encourage asking of questions M14. Prompt identification and seek available social support T1.1 Goal Setting (behavior) T1.3 Goal Setting (outcome) T1.4 Action planning T1.5 Review behavioral goals T1.7 Review Outcome Goals T3.2 Social Support (Practical) T3.3 Social Support (Emotional) T6.2 Social comparison T6.3 Information on others’ approval. T13.1 Identification of self as role model T13.5 Identity associated with changed behavior

In addition to the web-based sessions, participants will have access to a website that includes materials that support the content of each session. This includes worksheets, slide decks and recorded versions of the sessions, a menu of physical activities suitable for parents and children, and further practice materials.

Between sessions, participants will receive SMS text messages that ask them to provide written feedback on their progress; offer advice, suggestions, or reminders; or prompt reflection on their motives for physical activity. Parent participants will also be granted access to a moderated web-based chat forum (WhatsApp group [Meta Facebook, Inc]) in which parents can share their experiences with the sessions and provide and receive social support from other participating parents. Intervention materials are available on the internet [40].

#### Waitlist Control Group

Participants assigned to the waitlist control group will complete the same data collection procedures as the immediate intervention group but will not be required to undertake any alternative intervention tasks during the data collection period. After the data collection period, participants in the waitlist control group will be invited to receive the intervention and accompanying materials.

### Outcomes

#### Overview

Measures of psychological constructs will be assessed using multi-item scaled survey measures, while leisure-time physical activity is to be assessed using self-reported surveys and observationally through accelerometer measurements. All items were translated into Finnish by native speakers and piloted on a sample of 8- to 12-year-old Finnish children and their parents. Full measures are available on the internet [40].

#### Primary Outcomes (Physical Activity and Sedentary Time)

Self-reported physical activity and time spent in sedentary activities for both parents and children will be assessed using the Godin-Shepard leisure time exercise questionnaire [41], where participants will be required to report the number of occasions they engaged in light, moderate, and vigorous physical activity for 15 minutes or longer. Sedentary time will be measured using 2 items per participant, targeting weekdays and weekend days separately (eg, “In the past 7 days, how much time did you spend sitting during a typical weekday after school or weekend day?”) [42]. Items are scored on a sliding scale from “no time” upwards in increments of half an hour.

#### Secondary Outcomes

##### Device-Measured Physical Activity

Physical activity will also be assessed using a hip-worn triaxial accelerometer (Hookie AM20; Traxmeet Ltd). Each dyad will be mailed 2 accelerometers, detailed instructions on wearing the device, and a diary for recording when they wore the accelerometer, how they commuted to school or work, and any events that may have inhibited accurate data (eg, missed days and exercise done without the device). Raw data from accelerometers will be processed using the *GGIR* package in R software (R Foundation for Statistical Computing) [43], with outcome scores provided as the amount of time spent engaged in sedentary behavior, light physical activity, moderate physical activity, and vigorous physical activity.

##### Autonomy-Supportive Parenting

Autonomy-supportive parenting practices will be assessed using a 4-item questionnaire based on measures used in a previous study [44], with responses provided on 5-point scales (1=strongly disagree to 5=strongly agree).

##### Perceived Autonomy Support

Perceived autonomy support will be assessed using the perceived autonomy support scale for exercise settings [45]. For children, the scale refers to autonomy support from parents, while for parents, the scale makes reference to autonomy support received from family. All items are scored on a 5-point Likert scale (1=strongly disagree to 5=strongly agree).

##### Autonomous and Controlled Motivation

Autonomous and controlled motivation for both parents and children is assessed using 4 items each [46], with responses provided on 5-point scales (1=strongly disagree to 5=strongly agree).

##### Attitude

Attitude toward engaging in physical activity is assessed using 3 items with a common stem [44,47], with responses provided on a 5-point semantic differential scale (eg, unenjoyable [1] to enjoyable [5]).

##### Subjective Norms

Subjective norms in parents are assessed using 3 items referring to important others [44,47]. Children answered similar items, but in specific reference to family and friends separately. All items are scored on a 5-point Likert scale (1=strongly disagree to 5=strongly agree).

##### Perceived Behavioral Control

Perceived behavioral control is assessed in both parents and children using 2 items [44,47], with responses provided on 5-point scales (1=strongly disagree to 5=strongly agree).

##### Intention

Intention to engage in leisure time physical activity is assessed in both parents and children through 3 items [44,47], with responses provided on 5-point scales (1=strongly disagree to 5=strongly agree).

##### Self-Monitoring

Self-monitoring toward physical activity is assessed using 2 items [44,48], each scored on a 5-point Likert scale (1=strongly disagree to 5=strongly agree).

##### Habit

Habits are assessed using the 4-item automaticity subscale of the self-reported habit index [49,50], with responses provided on 5-point scales (1=strongly disagree to 5=strongly agree).

##### Acceptability

For participants in the immediate intervention group, the postintervention (3 months) web-based questionnaire will include survey items assessing the accessibility and feasibility of intervention procedures. Participants will also be invited to attend a 45-minute web-based exit interview to explore participant perceptions of the intervention content and possible improvements for future implementation.

### Data Analysis

Hypotheses will be tested using R software. All analyses will initially be performed as intention-to-treat, and per-protocol analyses will also be undertaken for comparison. Patterns of missing data will be explored using the Little missing completely at random test. Missing data in the final analysis will be inferred using full-information maximum likelihood analysis. We will test the efficacy of the intervention on our primary outcome, self-reported leisure-time physical activity, using an iterative series of generalized linear models. Independent variables will include time, intervention condition, demographic covariates (eg, start date, gender, and age), delivery group clustering, within-dyad clustering, and person-intervention theory fit *P*Δ [51]. Each variable group will be added in a subsequent iteration of the model, and model fit statistics will be examined at each iteration. This process will be repeated for each secondary outcome variable (perceived autonomy support, autonomous motivation, attitude, subjective norm, perceived behavioral control, action planning, coping planning, self-monitoring, and behavioral automaticity).

We also intend to assess the effect of theory-based mediators on change in physical activity using a path model. Specifically, we aim to assess whether the effects of intervention conditions on change scores in physical activity outcomes (both primary and secondary) are mediated by change scores in each of the psychological constructs targeted by the intervention (ie, perceived autonomy support, autonomous motivation, attitude, subjective norm, perceived behavioral control, action planning, coping planning, self-monitoring, and behavioral automaticity).

### Ethical Considerations

All study procedures have been approved by the University of Jyväskylä Human Sciences Ethics Committee (statement number 806/13.00.04.00/2023). All parents interested in participating in the study will be presented with detailed information about the intervention, potential risks to participants, the right to withdraw, and data security arrangements. Parents will have the chance to read this information and ask questions of the research team before providing their informed consent to participate. Data will be stored on secure cloud-based servers hosted by the University of Jyväskylä consistent with our data archiving and storage management plan, compliant with university guidelines. At the conclusion of data collection, participants’ physical activity and data on psychological measures at each measurement point will be matched using pseudonymized codes and deidentified to the greatest extent possible. Participants will not be offered any financial or other compensation for their participation.

## Results

The project team received final notification of research funding approval for the current project from the Finnish Ministry of Education and Culture, Sport Science Funds, in March 2022 (PROJECT 350904), and the trial has been preregistered on ClinicalTrials.gov (ID 806/13.00.04.00/2023). Enrollment into the trial commenced on September 20, 2023. Enrollment is scheduled to continue until March 2024, with the final collection of follow-up data scheduled for December 2024.

We expect that the research will provide valuable formative evidence for the efficacy of a theory-driven family-based physical activity intervention strategy. Further, as the proposed trial includes open materials and tests of the theory-driven mechanistic effects that may encourage behavior change, this research may also serve as a valuable stepping stone to the development of more large-scale, low-cost interventions for family behavior change.

## Discussion

### Overview

This protocol presents a randomized controlled trial aiming to increase physical activity levels in inactive parent-child dyads within Finland, based on the integrated behavior change model. We hypothesize in this protocol that both parents and children will show increased levels of physical activity, our primary outcome variable, both at the immediate and 3-month postintervention stage, relative to a waitlist control group. Further, we hypothesize we will observe similar changes in the trial’s secondary outcomes, the psychological constructs of the integrated behavior change model (ie, autonomy support, autonomous motivation, attitude, subjective norm, perceived behavioral control, self-monitoring, intentions, and habit), in the intervention group relative to the waitlist control group.

### Potential Findings and Implications

Children transitioning into adolescence have shown a sharp decline in activity levels [3], while parents are generally less active than similar adults without children [8]. Thus, both populations individually represent valuable targets for intervention. Recognizing this, governments and health departments have recommended behavioral interventions to promote physical activity participation in both groups. However, beyond strategies to influence physical activity in either children or parents separately, research indicates that parents and children likely have a noteworthy influence on each other’s physical activity behaviors and beliefs [6,7,9]. Thus, the delivery of interventions in family contexts, such as parent-child dyads, represents a potentially highly valuable strategy to promote physical activity participation in both populations. This is supported to a degree in meta-analysis, as dyadic interventions encouraging an active lifestyle demonstrated slightly larger effects than those targeting individuals [10]. Yet, such interventions remain relatively rare compared to more traditional, individual-targeted programs, particularly those that are based on behavioral theory which may contribute to their efficacy and relevance to health sciences overall [10].

This study tests a theory-based family behavioral intervention aimed at promoting change in physical activity participation in parent-child dyads. The intervention aims to foster autonomous motivation, enhance social support, and reduce perceived barriers to exercise in an atmosphere that is accepting and open. The intervention will make a unique contribution to practice and theory. Given the low levels of physical activity participation in adult and child populations, demonstrating the efficacy of web-based behavioral intervention in increasing physical activity that is both replicable and potentially scalable will make a valuable contribution to practice in health care contexts. In this research, we aim to enhance the potential usefulness of this intervention in the context of informing refined, scalable interventions based on results with the use of open materials and data, including intervention content and delivery guides. Further, from a scientific perspective, the application of a theory-based intervention developed in line with current practice intervention guidelines presents a potentially valuable test of mechanistic effects presented in the integrated behavior change theory and its component models [12-15], identifying the “active ingredients” of the intervention that are associated with desired outcomes. That is, while the integrated behavior change theory has been supported in several correlational studies [22,23,52], such research only provides an indication of the likely variables most important in determining behavior and can by their correlational nature not be used for any assertions of direction or causality. Thus, a key contribution of this intervention is assessing not only whether the program is successful in changing behavior, but also in assessing which target constructs mediate the effects of the intervention on behavior change and may therefore be most valuable when refining current strategies or developing new programs.

### Limitations

Beyond the expected value presented by the research, it is also important to note that the trial faces some expected and inherent challenges and limitations. For example, as the intervention does not include any reward or payment to participants beyond the benefits of the intervention itself, it is likely that parents who consent to enroll themselves and their child in the program will already be at least somewhat motivated to change their physical activity behavior. Such an issue has been noted in the previous parent-for-child interventions [53]. If this is the case, it is likely that the intervention effects will not be as strong as expected, as already motivated participants possess a lesser degree of potential for change than might be expected in families with unmotivated parents. While this nonetheless poses a challenge to the intervention, it is important to note that motivation or knowledge of the need for physical activity is commonplace [9,54], even as actual activity levels remain low. As the strategies used in this intervention include training parents in autonomy support rather than controlling strategies that may inhibit their child’s autonomy and thus harm the development of active lifestyles [20,55], as well as strategies to bridge the intention-behavior gap, this trial still has bona fide value in targeting this key population. However, the problem of accessing and enrolling unmotivated families into intervention programs remains a concern for behavior change research.

### Conclusions

Given the generally low levels of physical activity in Finnish parents and children, there is a notable need for intervention strategies aiming to encourage an active lifestyle in these populations. This protocol presents an upcoming randomized control trial based upon the integrated behavior change model, which aims to use a series of web-based, theory-based behavior-change strategies delivered to both parents and children as a dyadic program. In doing so, the proposed trial aims to extend upon current literature in several key aspects. First, by targeting parents and children as a dyad, the proposed study aims to add to the available literature on whether physical activity behavior change programs may be more efficacious when targeting the family unit, rather than parents or children individually. Second, as this study uses a theory-based design, testing change in both physical activity and related psychological constructs, the trial also offers an opportunity to test which beliefs and psychological factors are most associated with concomitant change in physical activity. These data, combined with the trial’s open materials, may thus serve as a valuable stepping stone to the development of more large-scale, low-cost interventions for family behavior change.

## Appendix

Multimedia Appendix 1 Peer review report from the Finnish Ministry of Education and Culture.
