# Supplementary material for: A Web-Based Physical Activity Promotion Intervention for Inactive Parent-Child Dyads: Protocol for a Randomized Controlled Trial
Source: JMIR Res Protoc. 2024 Mar 21;13:e55960. doi: 10.2196/55960 (PMC10995784; doi:10.2196/55960)
Supplement: Multimedia Appendix 1 [file resprot_v13i1e55960_app1.pdf]

Date	28.01.2022	Overall rating	5
Panel	Life05_22		
Experts	Colin Moran, Elizabeth Pike, Elling Bere, Jostein Hallén, Karen Birch, Samantha Winter, Sarah Astill, Stéphane Baudry, Thomas Bredahl, Willem van Mechelen		
Support reviews			
Application No.	350904		
Call	Funding for sport science research projects from Ministry of Education, Science and Culture 25.08.2021 - 30.09.2021		
Applicant	Martin Hagger		
Research topic	A collaborative theory-based intervention to promote physical activity among sedentary parents and their children (ProAct)		

1 Quality of research described in the plan

1.1 Scientific quality, novelty and innovativeness of research

Significance of project; objectives and hypotheses; ambitiousness and state of the art of
objectives (possible novel concepts and approaches or development across disciplines);
scientific impact of research; potential for breakthroughs or exceptionally significant outcomes;
etc.

This project will develop and evaluate a theory-based intervention to promote sustained physical activity participation among sedentary parents and their children. It is a well written proposal. It is based on state-of-the-art science, e.g. regarding use of theory in intervention development. Objectives, research questions and hypotheses are very clear and sound as well. The topic of increasing physical activity of sedentary parents and children is of high importance.
However, the study as such is not really innovative or novel. These kind of theory-driven studies have been performed before.
Still, if successful, the study outcome will have major societal (Public Health) impact, as the results must be translated into practice guidelines. The project will certainly have scientific impact.

1.2 Implementation of research plan

Feasibility of project (bearing in mind extent to which the proposed research may include high risks); materials, research data and methods; human resources and management of research tasks; research environment including research infrastructures; identified potential scientific or methodological problem areas and mitigation plan; etc.

Methods are very well described. It is clear what will be done, and everything make sense. It is a lot of work, but it absolutely appears feasible. Measurements are well chosen and the rationale for choosing them are discussed. Good discussion on sample and power calculation. Smart to use the panel company to recruit. Scientifically this is state-of-the-art.
Still, the proposal is not totally convincing that an intervention consisting of 8 intervention 'steps/modules' will lead to sustained physical activity behaviour change (i.e. beyond 12 months after baseline). Will the proposed relatively minimal intervention of short duration be able to counteract in a sustained way the upward behavioural pressures that are put on a child by the 'toxic environment' we live in?
Also, it was somewhat unclear what the inclusion criteria is regarding level of physical activity. How sedentary must the parent and child(ren) be, and is it both that has to be sedentary?

2 Competence of applicant(s), quality of research collaboration

2.1 Competence of applicant(s) and complementary expertise of applicant's research team (project personnel)

Merits and scientific expertise of applicant in terms of project implementation; complementary expertise of applicant's research team (i.e. project personnel directly working/funded for the project); competence of applicant(s) in terms of supervising PhD candidates or postdoctoral researchers; support for researcher training within project; etc.

The track record of this applicant is outstanding. This project builds very well on previous work from his group. Research group is very well selected. Together they are very capable to conduct this research.

2.2 Significance of research collaboration and researcher mobility

Significance of national and/or international research collaboration (i.e. collaborators engaged in the project with their own funding) including complementary expertise and research environment of collaborators in terms of project implementation; significance of planned mobility for implementation of research plan and researcher training; etc.

Several well selected strong international collaborators with added (as well as overlapping) expertise to the project. Some mobility visits are included.

3 Responsible science

3.1 Has the applicant considered the following aspects of responsible science properly in the application?

3.1.1 Research ethics

Yes

3.1.2 Promotion of equality and non-discrimination within project or in society at large

Yes

3.1.3 **Open access of research publications**

Yes

3.1.4 **Data management and open access to data**

Yes

3.2 **Comment on responsible science, societal effects and impact**

3.2.1 **Provide further comments if responsible science aspects above (3.1.1 – 3.1.4) have not been properly considered.**

3.2.2 **Additional comments on societal effects and impact**

You are also encouraged to comment on the societal effects and impact, including principles of sustainable development.

4 Overall assessment and rating

4.1 **Main strengths and weaknesses of project**

Please select major strengths and weaknesses of the application. Give justifications for the selection in sub-item 4.2.

4.1.1 **Main strengths (select all relevant aspects):**

☑ scientific quality
☐ innovativeness and novelty value
☑ impact within scientific community
☑ feasibility of research plan
☐ significance and added value of consortium (if applicable)
☑ competence of applicant/s in terms of project implementation
☑ complementary expertise of research team (in terms of project implementation)
☑ significance of collaborative networks in terms of project implementation
☑ researcher training including researcher mobility

4.1.2 **Main weaknesses (select all relevant aspects):**

☐ scientific quality
☐ innovativeness and novelty value
☐ impact within scientific community
☐ feasibility of research plan
☐ significance and added value of consortium (if applicable)
☐ competence of applicant/s in terms of project implementation
☐ complementary expertise of research team (in terms of project implementation)
☐ significance of collaborative networks in terms of project implementation
☐ researcher training including researcher mobility

4.2 **Justifications and comments**

Please justify the selections above by briefly describing the main strengths and weaknesses of the application.

Strengths:

- The proposal is from a technical perspective written in a state-of-the-art manner.
- The project is well planned.
- The topic is important.
- The research group is strong with strong collaborations.

Weaknesses:

- It lacked a bit of innovativeness.
- It was somewhat unclear with the inclusion criteria.
